# Use of dynamic microsimulation to predict disease progression in patients with pneumonia-related sepsis

**DOI:** 10.1186/cc5942

**Published:** 2007-06-14

**Authors:** Görkem Saka, Jennifer E Kreke, Andrew J Schaefer, Chung-Chou H Chang, Mark S Roberts, Derek C Angus, the GenIMS Investigators

**Affiliations:** 1 Department of Industrial Engineering, University of Pittsburgh, 3700 OHara St., 3700 Benedum Hall, Pittsburgh, PA 15261, USA; 2 Section of Decision Sciences and Clinical Systems Modeling, Department of Medicine, Division of General Internal Medicine, University of Pittsburgh, 200 Meyran Ave., Suite 200, Pittsburgh, PA 15213, USA; 3 The Clinical Research, Investigation, and Systems Modeling of Acute Illness (CRISMA) Laboratory, Department of Critical Care Medicine, University of Pittsburgh, 3550 Terrace St., 600 Scaife Hall, Pittsburgh, PA 15261, USA

## Abstract

**Introduction:**

Sepsis is the leading cause of death in critically ill patients and often affects individuals with community-acquired pneumonia. To overcome the limitations of earlier mathematical models used to describe sepsis and predict outcomes, we designed an empirically based Monte Carlo model that simulates the progression of sepsis in hospitalized patients over a 30-day period.

**Methods:**

The model simulates changing health over time, as represented by the Sepsis-related Organ Failure Assessment (SOFA) score, as a function of a patient's previous health state and length of hospital stay. We used data from patients enrolled in the GenIMS (Genetic and Inflammatory Markers of Sepsis) study to calibrate the model, and tested the model's ability to predict deaths, discharges, and daily SOFA scores over time using different algorithms to estimate the natural history of sepsis. We evaluated the stability of the methods using bootstrap sampling techniques.

**Results:**

Of the 1,888 patients originally enrolled, most were elderly (mean age 67.77 years) and white (80.72%). About half (47.98%) were female. Most were relatively ill, with a mean Acute Physiology and Chronic Health Evaluation III score of 56 and Pneumonia Severity Index score of 73.5. The model's estimates of the daily pattern of deaths, discharges, and SOFA scores over time were not statistically different from the actual pattern when information about how long patients had been ill was included in the model (*P *= 0.91 to 0.98 for discharges; *P *= 0.26 to 0.68 for deaths). However, model estimates of these patterns were different from the actual pattern when the model did not include data on the duration of illness (*P *< 0.001 for discharges; *P *= 0.001 to 0.040 for deaths). Model results were stable to bootstrap validation.

**Conclusion:**

An empiric simulation model of sepsis can predict complex longitudinal patterns in the progression of sepsis, most accurately by models that contain data representing both organ-system levels of and duration of illness. This work supports the incorporation into mathematical models of disease of the clinical intuition that the history of disease in an individual matters, and represents an advance over several prior simulation models that assume a constant rate of disease progression.

## Introduction

Each year in the USA 750,000 people develop severe sepsis, a systemic inflammatory response with acute organ dysfunction that occurs secondary to infection [1]. About one-third of patients die, making sepsis a major cause of mortality [2]. Because care for patients with sepsis is complex and many questions concerning the clinical course and treatment cannot be explored via randomized controlled trials, several investigators have applied mathematical modeling to examine the relationships between patient characteristics, disease progression, and outcomes. For example, Bauerle and coworkers [3] developed a stationary Markov model with three states (well, septic, and dead) to describe the course of disease in critically ill patients, to produce risk profiles of various patient groups, and to estimate age-specific and sex-specific survival rates. In this model, the transitions were independent of time and did not incorporate information regarding the patient's duration of disease. Using a similar structure, Rangel-Frausto and colleagues [4] modeled the stages of sepsis (sepsis, severe sepsis, septic shock, and death), calibrated from a single center prospective cohort study. More recently, Clermont and coworkers [5] developed a microsimulation model that characterized patients admitted to the intensive care unit (ICU) in terms of their scores on the Sepsis-related Organ Failure Assessment (SOFA) [6]. The Clermont model first predicts the changes in component SOFA and total SOFA scores over time, and then it uses these data to predict death, transfer out of the ICU, or continued presence in the ICU. Although the model recognizes the nonconstant nature of transition probabilities, it does not include data concerning the clinical course of patients before they enter the ICU.

We describe the construction of a simulation model that uses a more detailed description of illness (represented by changes in both the total and component SOFA scores) and allows the progression of disease to depend upon the duration of illness and the recent history, namely whether the patient is improving, becoming worse, or remaining stable. The potential use of such a simulation model is broad, and it is more flexible than a standard statistical prediction rule. A standard prediction rule typically estimates the outcome as a function of initial variables, such as the likelihood of death given age, sex, level of illness, and so on. However, it is not capable of predicting an individual's actual course of disease. In contrast, a simulation model produces a virtual representation of each individual, their specific course through their disease, and eventual outcome, which can be used to investigate the potential effects of process of care or therapeutic interventions across the entire course of disease.

The purpose of this investigation is to provide a 'proof-of-concept' that the simulation technique can model individual patients whose aggregated disease course reproduces the rate of change of severity of illness, and the actual outcomes of a multicenter cohort of patients at risk for severe sepsis.

## Materials and methods

### Sources and types of data used

The data that we used to calibrate our model were derived from patients who were enrolled in the GenIMS (Genetic and Inflammatory Markers of Sepsis) study, which is a multicenter cohort study of patients with community-acquired pneumonia (CAP) who are at risk for severe sepsis. The research performed in the original study and subsequent model development was approved by the institutional review boards of the University of Pittsburgh and other participating universities and hospitals.

The GenIMS Study enrolled patients from 28 academic and community hospitals in southwestern Pennsylvania, Connecticut, southern Michigan, and western Tennessee. Patients were eligible for inclusion if they were older than 18 years and had a clinical and radiological diagnosis of pneumonia, following the criteria proposed by Fine and coworkers [7] They were subsequently excluded if they met any of the following criteria: transfer from another hospital, discharge from a hospital within the prior 10 days, occurrence of an episode of pneumonia within the prior 30 days, long-term use of mechanical ventilation, presence of cystic fibrosis or active pulmonary tuberculosis, admission for palliative care, previous enrollment in the study, incarceration, and pregnancy.

We used data describing each patient's demographic characteristics (age, sex, and race/ethnicity) and hospital stay (dates of admission, movement to or from a hospital ward, movement to or from an ICU, discharge, or death). The clinical detail contained in the GenIMS data also allowed us to estimate the progression of disease over time in terms of both the level of illness (as represented by the daily SOFA score) and the direction of progression (direction of change in SOFA score over time). The SOFA scores describe in quantitative terms the degree of organ dysfunction or failure, as defined by the Working Group on Sepsis-related Problems of the European Society of Intensive Care Medicine [6]. In the GenIMS study patients were considered to have severe sepsis if their SOFA score for any organ system was 3 or a 4, provided that the level of dysfunction in that organ system was not as severely impaired in the patient's pre-morbid state. When organ dysfunction data were missing (for instance, serum bilirubin), scores were imputed using an algorithm (Table 1) based on methods from previous sepsis studies [5,8].

**Table 1:** Handling of missing SOFA scores in the total sample, calibration sample, and validation sample

Type of missing SOFA data	Interpolation and extrapolation rules used to fill in missing data
Data have never been measured	Use the baseline SOFA value for every day
Data are missing between two known values	Linearly interpolate between the values
Data are missing before the first observation	Use the baseline SOFA value for every day until the first observation
Data are missing after the last observation and the patient died	Assign the highest SOFA score (4) to the last day. Linearly interpolate between the last observation value and the last day value
Data are missing after the last observation and the patient was discharged	Assign the baseline SOFA value to the last day. Linearly interpolate between the last observation value and the last day value
Data are missing after the last observation and the patient was still in the hospital at day 30	Carry the last observation forward

The total sample included 1,888 patients from the GenIMS (Genetic and Inflammatory Markers of Sepsis) Study. SOFA, Sepsis-related Organ Failure Assessment.

### Development of the model

**Figure 1:**
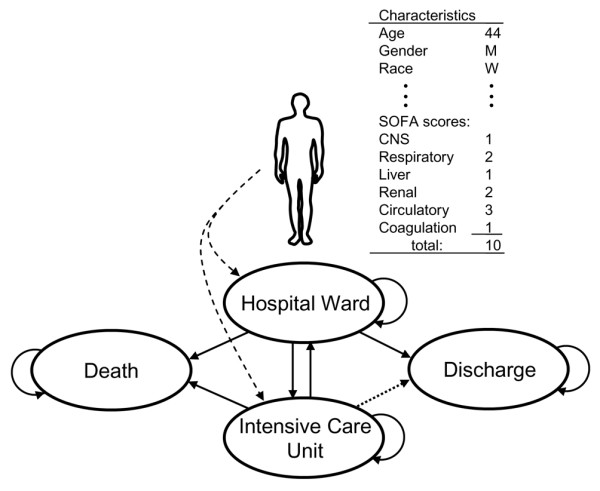
Basic structure of the simulation model. In the model, a patient with static and dynamic characteristics enters the hospital ward or intensive care unit (ICU). The patient could remain in the same location, move between the ward and ICU, die, or be discharged from the hospital. CNS, central nervous system; SOFA, Sepsis-related Organ Failure Assessment.

The purpose of the model is to simulate a cohort of patients whose disease progression represents the collective experience of the actual patients in the GenIMS cohort. Figure 1 describes the basic structure of the simulation. A patient is initially admitted to a hospital location (ward or ICU) and can either remain there or move from one hospital location to another until death or discharge, or until 30 days have elapsed. To create clinical progressions (called 'trajectories') in these simulated patients, the model has methods for generating patients and updating their health over time.

Using a modification of methods described in detail by Alagoz and coworkers [9], the model takes an actual patient's SOFA score history and decomposes it into a sequential series of overlapping three-day scores (hereafter called 'triplets') [9]. The purpose of the triplets is to represent successive days of illness by decomposing each patient's experiences into multiple, overlapping three-day examples of yesterday's SOFA score, today's SOFA score, and tomorrow's SOFA score. For example, assuming a real patient remained in the hospital for five days, the first triplet would consist of the patient's scores for the first, second, and third days; the second would consist of scores for the second, third, and fourth days; and the final triplet would consist of scores for the third, fourth, and fifth days.

To utilize as much of the GenIMS dataset as possible, we developed special cases for those patients who remained in the hospital for less than three days. If the patient stayed for only two days, then the triplet is made by duplicating the scores for the first day and assuming that the patient was stable the day before they came into the hospital. If a patient stayed in the hospital for only one day, then the triplet is made by replicating those data twice, reflecting the fact that we have no information regarding the direction of illness progression.

### Initial patient generation

To generate a cohort of patients that resembles at baseline the actual study cohort, the model generates a set of virtual patients by randomly selecting triplets from the set of triplets whose first day happened to be day one for an actual patient. Each virtual patient is then assigned the demographic data (age, sex, and ethnicity) associated with that first triplet.

### Disease progression

**Figure 2:**
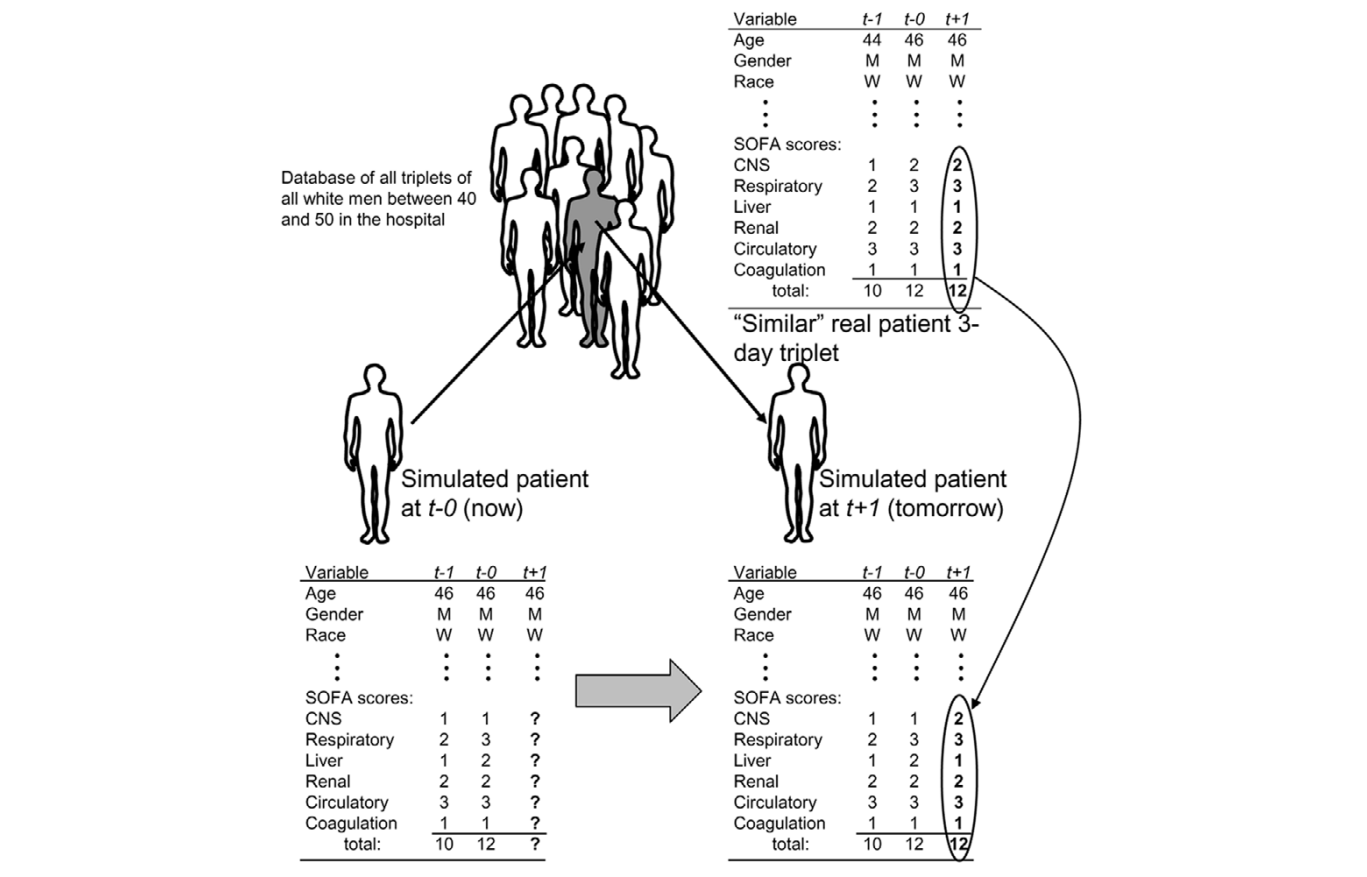
Empiric method for updating the patient's health. To update a model-generated patient's SOFA scores from one time to the next (from *t *- 0 to *t *+ 1), the model searches for a patient with similar characteristics at *t *- 0. The model finds the 'similar' patient's *t *+ 1 scores and uses them to represent the generated patient's *t *+ 1 scores. CNS, central nervous system; SOFA, Sepsis-related Organ Failure Assessment.

The method used to determine the progression of disease and future health status of the generated patients is illustrated in Figure 2. The figure describes a simulated 46-year-old white male with a current total SOFA score of 12 (composed of the component scores shown) who was slightly less sick the previous day, with a total SOFA score of only 10. The algorithm searches the set of all triplets derived from patients who are clinically 'similar' (defined below) to the generated patient, and randomly picks one of them. The model then uses that chosen patient's next day SOFA scores (labeled *t *+ 1 in the figure) to fill in the generated patient's SOFA scores for the next day. The model advances time by one day, and the generated patient's *t *+ 1 values become the current day values, and the process repeats itself. In addition to the SOFA scores, several other events are carried forward with each triplet, including the patient's location in the hospital (ward, ICU, or discharged) and whether the person is alive or dead. For example, if the patient represented by the chosen triplet died during the next time period, then the generated patient is considered to have died that next day as well. If the patient represented by the chosen triplet was transferred from the ICU to the ward or was discharged from the hospital, then the same event is recorded for the simulated patient.

### Determining clinical similarity

One of the major goals of this model development was to determine what level of clinical similarity is necessary in matching patients (which means choosing a triplet that is similar to the generated patient) to reproduce the events and longitudinal disease progression observed in the actual cohort. Several characteristics were assumed to be important by the GenIMS investigators. When searching the database of triplets, the model only searches among triplets that are derived from patients who are in the same age category (< 65 years, 65 to 80 years, and > 80 years), the same racial/ethnic category (white and African American), and the same location in the hospital (ward or ICU).

Several different methods were used to match patients based on the severity and duration of illness. The different methods were tested to assess which characteristics were most important in creating a set of generated clinical histories that most closely matched the real clinical histories. The model uses the SOFA score to represent severity of illness, and the model can match on either the total score (in which case the score must match exactly) or on the components of the score, which are central nervous system, respiratory, circulatory scores and the maximum of liver, renal, and coagulation scores. The component SOFA scores were aggregated into categories of 0, 1 to 2, and 3 to 4. Component scores were considered similar if they were in the same three-level category.

Whether the model matches on total or component SOFA, it can also match on whether the patient's health is improving, worsening, or staying the same by comparing whether the SOFA scores are declining, rising, or remaining stable compared with the prior day's score. Finally, the model can match on the duration of illness, as measured by length of stay aggregated into five categories (day 1, day 2, day 3, days 4 to 7, and day 8 and thereafter). In summary, the model can match on three different clinical characteristics: the severity of illness, measured by total or component SOFA score; the direction of illness progression, represented by whether the SOFA score is rising, declining, or remaining stable; and the duration of illness, represented by categories of length of stay in the hospital. The use of these three matching criteria produces eight possible algorithms for matching generated patients to real patient three-day segments (triplets): severity of illness (total or component SOFA), direction of illness progression (required or not), and duration of illness (required or not). Finally, the model does not allow the triplet that produced the most recent data to be matched to a triplet from the same patient to determine the next day's SOFA scores. This ensures that the model does not simply replicate the actual history of the patients in GenIMS, but rather each simulated history will be a combination of the histories of individuals who are generally similar.

There are criteria to handle certain special cases. If the model cannot find a triplet that is similar to the generated patient, then it expands the similarity criteria and allows the generated patient to be matched either to a triplet whose total SOFA score is 1 point lower or higher or to a triplet whose component SOFA score is 1 class lower or higher. If the generated patient cannot be matched to a triplet meeting either of these expanded criteria, then the model finds the next day values of the prior triplet that the generated patient matched and uses those. If the model does not find a match after using the expanded criteria and after trying to match to the last triplet's next day values, it removes the generated patient from the model.

Generated patients leave the simulation model if they die, if they are discharged, if their hospital stay exceeds 30 days, or if the model cannot predict their next day health status. The simulation model was created in C programming language.

### Statistical analysis

We used the model to predict three outcomes in patients hospitalized for up to 30 days: the number of discharges, number of deaths, and total SOFA scores of the patients in hospital. We used two methods to assess model performance. To compare these outcomes of simulations with those of the actual GenIMS dataset of patients, we used the Cressie-Read goodness-of-fit test, which is a special case (λ = 2/3) of a family of multinomial tests used to evaluate how well the observed frequencies fit with the expected frequencies [10]. This test determines whether the pattern of discharges and deaths over time in the simulated cohort is statistically different from the actual observed pattern. Second, to ensure that the model results are not representative of an idiosyncratic characteristic of the particular GenIMS database, we used standard bootstrapping techniques to assess the stability of the model results to variations in the input data. This is a robust extension of the 'split-halves' technique of a derivation and validation dataset. We simulated 100 different patient datasets, where each dataset is created by randomly picking 1,888 patients from the original patient data with replacement. Patients can be picked more than once and some patients may not be represented in each replicated dataset. The entire model runs of 50 replications are evaluated for each of the 100 datasets. We then computed the 95% confidence intervals on the mean of the 100 replications of the simulation. Stata version 9.0 (StataCorp, College Station, TX, USA) was used for the statistical calculations.

## Results

### Demographic and clinical characteristics

Of the 2,320 patients included in the GenIMS Study, 1,888 were eligible for inclusion in the model. The remainder were excluded because they were not hospitalized (291 patients), because the clinical team ruled out the presence of CAP (134 patients), or because the requisite data were missing (seven patients). Table 2 describes the baseline demographic and clinical characteristics of the 1,888 patients in the GenIMS dataset. Of these, most were elderly (mean age 67.7 years) and white (80.7%), and about half were women (48%). Most were relatively ill, as indicated by the following average scores at baseline: Charlson score of 1.9, Acute Physiology and Chronic Health Evaluation III score of 56, Pneumonia Severity Index score of 73, and SOFA score of 2.3. The specific etiology of pneumonia was available in 375 (16%) patients, and Gram-positive infections accounted for the majority of cases of pneumonia (*n *= 251). Almost 16% required intensive care, and 6.5% died within 30 days. Patients developing severe sepsis amounted to 31.2%, and of those 26.91% died within 90 days. Out of a total 13,820 patient-days, the algorithm used to fill in missing SOFA scores was used to impute 3,788 (27.41%) respiratory, 5,896 (42.66%) coagulation, 11,820 (85.53%) liver, 55 (0.40%) central nervous system, 3,959 (28.64%) renal, and 193 (1.40%) cardiac scores.

**Table 2:** Baseline demographic and clinical characteristics of the GenIMS cohort

Characteristic	Full GenIMS cohort (*n *= 2,320)	Simulation model cohort (*n *= 1,888)^a^
Age (years; mean ± SD)	65.6 ± 18.1	67.77 ± 16.80
Sex (% female)	47.70	47.98
Race (Caucasian/black [%])	79.20/16.30	80.72/15.73
Etiology (*n *[%])		
Bacterial pneumonia		
Gram positive only	251 (10.80)	237 (12.55)
Gram negative only	58 (2.50)	54 (2.86)
Mixed Gram positive and negative	22 (1.00)	21 (1.11)
*Chlamydia *or *Legionella *cultures	6 (0.30)	6 (0.32)
Other	38 (1.60)	37 (1.96)
Unknown	1,945 (83.80)	1,533 (81.20)
Charlson score (mean ± SD [% score = 0])	1.78 ± 2.16 (31.64)	1.93 ± 2.21 (27.54)
Admitted to hospital (*n *[%])	2,029 (87.50)	1,888 (100)
Admitted to hospital and pneumonia confirmed (*n *[%])	1,895 (81.70)	1,888 (100)
LOS (days; mean ± SD [median])	6.55 ± 5.10 (5)	7.26 ± 5.02 (6)
Admitted to ICU (%)	14.70	15.94
LOS in ICU (days; mean ± SD [median])	5.37 ± 5.04 (4)	5.53 ± 5.22 (4)
APACHE score day 1 (mean ± SD)	53.07 ± 20.30	56.23 ± 17.90
PSI time 0 (mean ± SD)	83.25 ± 34.25	73.53 ± 43.68
PSI I and II (≤70; %)	37.70	42.74
PSI III (71 to 90; %)	22.37	20.55
PSI IV (91 to 130; %)	30.14	27.60
PSI V (>130; %)	9.79	9.11
PSI day 1 (mean ± SD)	95.11 ± 40.41	100.16 ± 38.06
PSI I and II (≤70; %)	29.01	22.14
PSI III (71 to 90; %)	19.18	20.87
PSI IV (91 to 130; %)	33.79	37.34
PSI V (>130; %)	18.02	19.65
SOFA score day 1 (mean ± SD [% score = 0])	2.3 ± 1.92 (10.78)	2.33 ± 1.91 (12.50)
CNS organ failure, defined by the SOFA score (%)	5.22	5.93
Respiratory organ failure, defined by the SOFA score (%)	15.73	17.32
Liver organ failure, defined by the SOFA score (%)	0.52	0.64
Renal organ failure, defined by the SOFA score (%)	15.39	17.43
Circulatory organ failure, defined by the SOFA score (%)	3.58	3.97
Coagulation organ failure, defined by the SOFA score (%)	1.21	1.22
Discharged alive (%)	95.86	94.65
Severe sepsis subset mortality by day 90 (%)	25.51	26.91
In-hospital (%)	25.43	26.64
Mortality (%)		
30 day	6.12	6.46
60 day	8.35	9.16
90 day	10.36	11.28

^a^See section on demographic and clinical characteristics for explanation of patient exclusions from overall GenIMS database. APACHE, Acute Physiology Age and Chronic Health Evaluation; CNS, central nervous system; ICU, intensive care unit; LOS, length of stay; PSI, Pneumonia Severity Index; SD, standard deviation;

### Predictions of outcomes

**Figure 3:**
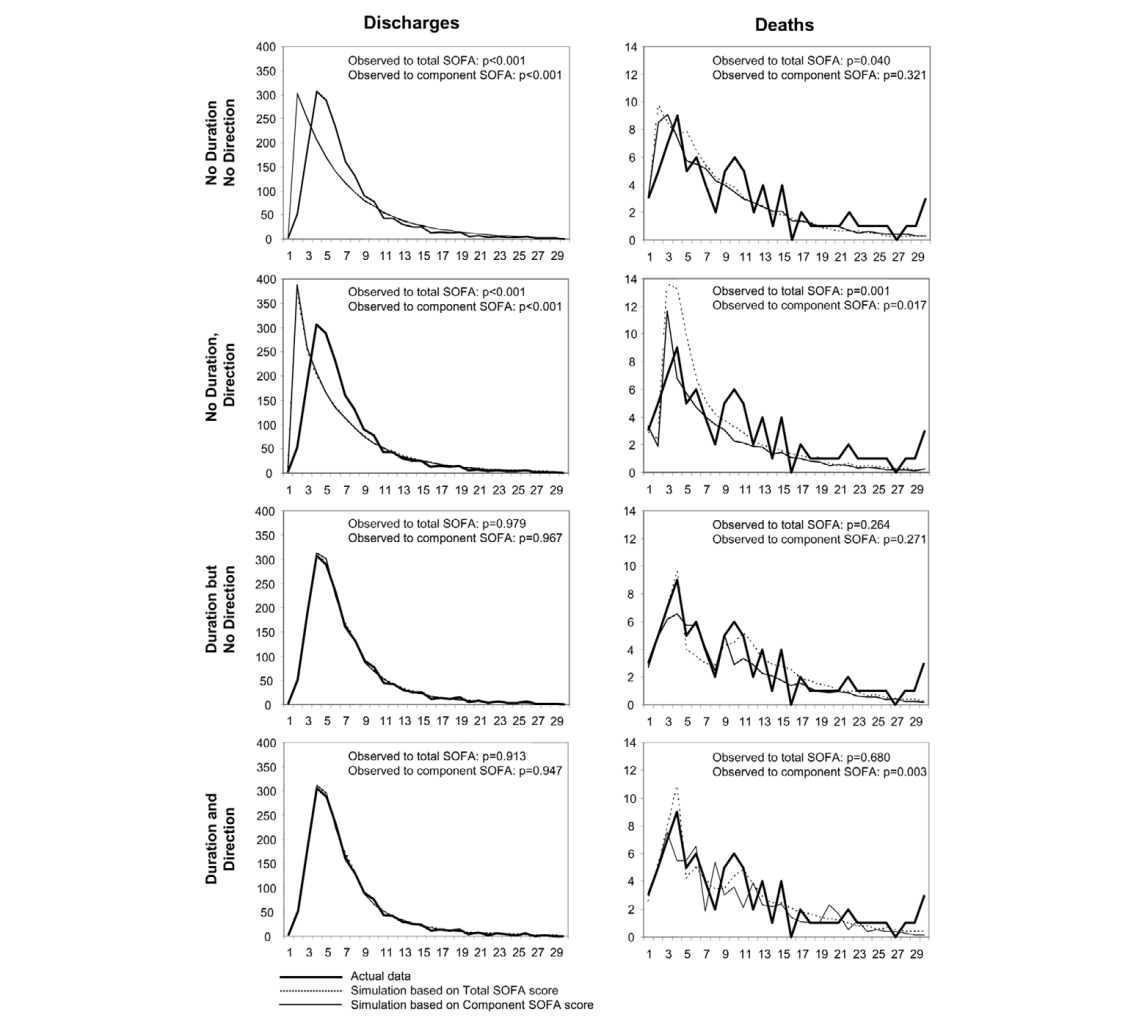
Predicted and actual (observed) numbers of discharges and deaths per day during hospitalization. The similarity criteria used for the predictions are least restrictive at the top of the figure (not matching on both duration in the hospital and direction of illness progression) and most restrictive at the bottom (matching on duration in the hospital and direction of illness progression). Simulated values are the average of 100 replications of the simulation. SOFA, Sepsis-related Organ Failure Assessment.

Figure 3 shows the model's ability to predict discharges and deaths using eight different algorithms matching patients according to level of component or total SOFA score, direction of change in SOFA score, and duration of illness. When the model was required to match on all of these criteria, the model closely predicted the pattern and number of discharges and deaths that occurred within 30 days. The GenIMS study recorded 1,787 actual discharges; the model predicted between 1,779 and 1,804 discharges, depending upon the algorithm used to match similar patients. There were 85 actual deaths, and the model predicted between 62 and 84, again depending upon the algorithm. In addition to predicting the number of events, a simulation model can predict the pattern of events over time.

As Figure 3 demonstrates, the model's ability to predict when deaths and discharges occur over time varies. In general, the more restrictive the criteria, the more closely does the model predict actual experience, although inclusion of duration of illness in the model had a greater impact than did inclusion of direction of illness progression. For example, when the algorithm does not include information on the duration of illness or the direction of progression (top left panel of Figure 3), the predicted pattern of discharges is statistically significantly different from the observed pattern (*P *< 0.001). When the algorithm incorporates information on the duration of illness and the direction of progression (bottom left panel in Figure 3) the pattern of predicted discharges is virtually indistinguishable from the actual (*P *= 0.91 to 0.95). The model was better able to predict death (but not discharge) when it used component SOFA scores than when it used total SOFA scores. Significance tests showed that the model's predicted distributions of discharges and deaths over time differed significantly from the actual distributions when no time stratification was used in the model, but they did not differ significantly when time stratification was used in the model. This indicates that the transitions that characterize sepsis are time dependent.

**Figure 4:**
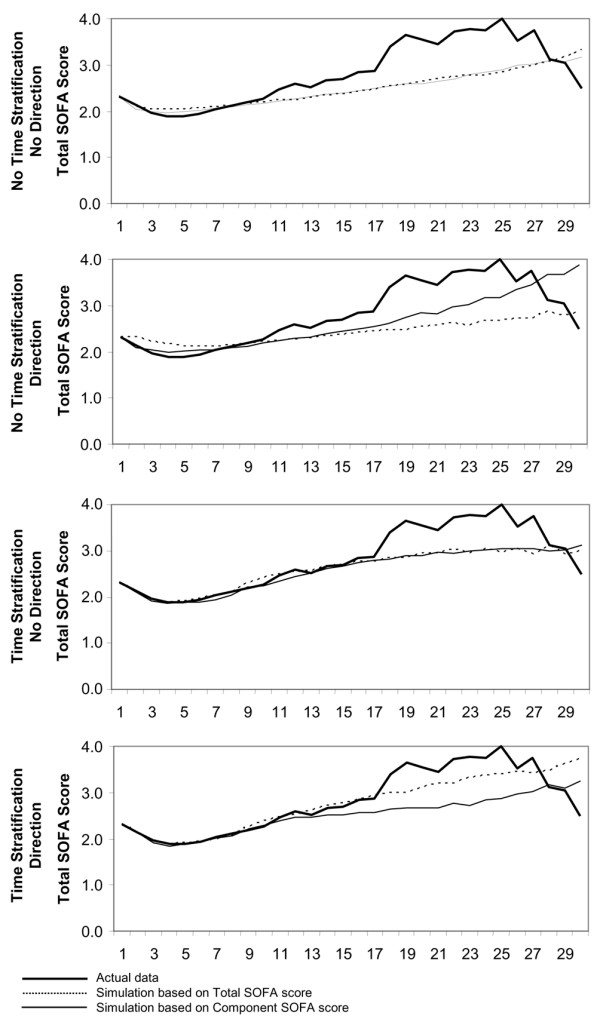
Predicted and actual (observed) daily average total SOFA score of patients in the hospital. The similarity criteria used for the predictions are least restrictive at the top of the figure (not matching on both duration in the hospital and direction of illness progression) and most restrictive at the bottom (matching on duration in the hospital and direction of illness progression). Simulated values are the average of 100 replications of the simulation. SOFA, Sepsis-related Organ Failure Assessment.

Figure 4 shows the model's ability to predict daily average total SOFA scores of patients who remained in the hospital. As was the case with the model's predictions of discharges and deaths, the model's predictions of scores most closely matched the actual scores when the model matched patients with respect to the length of time in the hospital, the level of illness (as measured by total or component SOFA scores), and the direction of illness. Although the inclusion of a measure of length of time in the hospital generally improved the prediction of the average SOFA scores of the surviving patients, the inclusion of a measure of whether the patients' health was improving or worsening (matching on the most recent direction of change in SOFA score) improved the prediction of the general level of illness to a greater degree than it improved the prediction of death or discharge.

**Figure 5:**
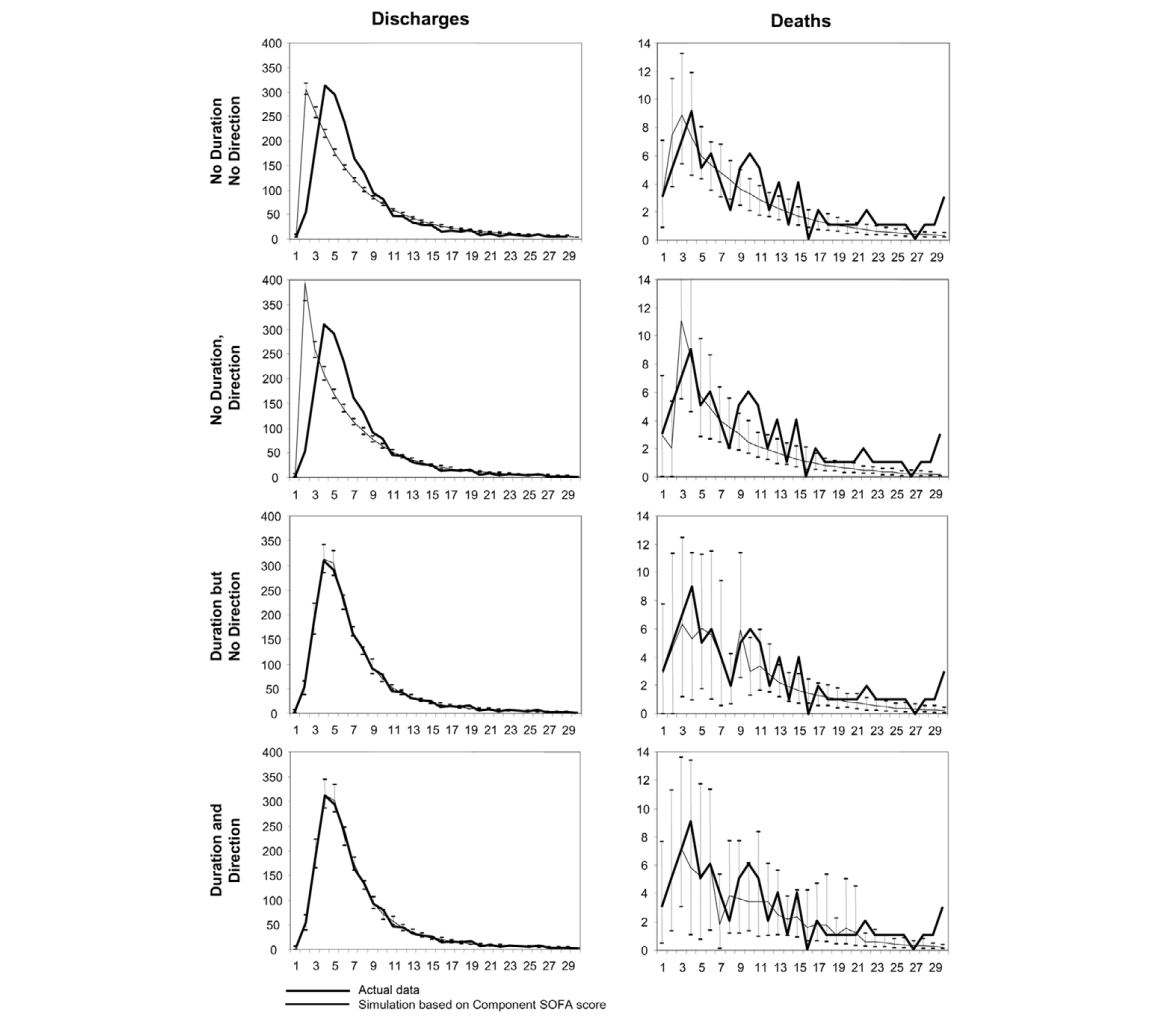
Bootstrap validation of the model results. The model was re-evaluated on 100 bootstrapped samples of 50 replications under each of the similarity criteria shown in Figure 3. Only results of the simulations using component SOFA scores are shown. Empiric 95% confidence limits around the predicted number of discharges or deaths each day are constructed from the distribution of simulated discharges or deaths on each day of the simulation. The results indicate that the model results are relatively stable to random fluctuations in the data that were used to calibrate it, and confirm the finding that duration of disease is more important in predicting overall outcome than the instantaneous direction of progression of disease.

The results of the bootstrap validations for the prediction of deaths and discharges are presented in Figure 5. When the model is calibrated from repetitive random samples of the original GenIMS data, and using only the component SOFA scores for simplicity of presentation, the number of simulated deaths and discharges on any given day varies within the 95% confidence limits shown. The figure demonstrates that the model is relatively stable to random fluctuations in the data used to calibrate it, although it is difficult to recreate accurately small numbers of events such as three or four deaths out of thousands of people. Hence, there are relatively wider confidence limits surrounding the model prediction of deaths than of discharges. The bootstrap validations also confirm the basic finding that the accurate prediction of the clinical course requires information regarding how long the patient has been ill. For example, when the model excluded information on the duration of illness and direction of progression of illness, the actual number of discharges were contained within the 95% confidence limits of the model prediction for only 13 out of 30 days (43.3%; upper left panel of Figure 5), but when information on both of these parameters was included the actual number of discharges were contained within the 95% confidence limits of the model prediction on 24 of 30 days (80%; lower left panel of Figure 5). Furthermore, all of the days in which the actual number of discharges was not contained within the confidence limits of the model occurred after 15 days of hospitalization, when the number of discharges was very low.

## Discussion

We used data from a large, multicenter trial to develop and evaluate an empiric simulation model that represents the time course of individual patients who are admitted to the hospital with CAP and are at risk for sepsis and multisystem organ failure. Our model expands on previous models in several ways. Like the Clermont model [5], our model represents individual patients and their SOFA scores over time and thereby adds a degree of clinical detail that is not seen in state-based Markov models [4]. In addition, our model simulates the longitudinal progression of component SOFA scores dependently, maintaining the inherent statistical associations found in the progression of sepsis across organ systems. The model incorporates data from each patient's disease history and represents the patient's changing health over time, not only as it relates to the patient's previous health state but also as it relates to the patient's length of stay in the hospital. Thus, it mitigates the 'lack of memory' assumptions inherent in standard stationary Markov models.

Two of our findings are of particular interest. The first is that when the model uses the level of illness as measured by SOFA scores to predict the course of hospitalized patients with CAP, the predictions are better if information is added regarding how long each patient has been in the hospital than if information is added regarding the acute (previous day) determination of whether the patient is improving or deteriorating. This observation reinforces a clinical intuition that a patient with a given level of organ dysfunction on day 3 is very different from a patient with that same level of organ dysfunction on day 15. The second is the finding that the predictive ability of the component and total SOFA scores is not equivalent. Of the two types of SOFA scores, the component scores appear to be better predictors of progression in the absence of information regarding how long the patient has been in the hospital, whereas the total scores appear to be better predictors in the presence of this information.

Our modeling technique has several limitations. The most important is that it is highly data intensive, and the division of the dataset into groups of patients who are 'similar' in terms of a series of criteria rapidly renders the membership in the individual groups small. Subgrouping by age, race/ethnicity, ICU status, duration in hospital, and SOFA score produced 240 groups with an average group size of about 50 triplets, although some specific combinations occurred much more frequently than others. Further investigations that use newer statistical techniques to predict multiple correlated data over time will be undertaken to address this data limitation. The etiology of pneumonia was determined in 16% of GenIMS patients, and it is possible that specific etiologies may predict outcome. However, these results are similar to previous clinical trials [11] and multicenter observational studies. For example, an etiology was confirmed in only 5.7% of cases in the PORT (Pneumonia Patient Outcomes Research Team) study conducted by Fine and coworkers [12]. Similarly, in a recent study by Metersky and colleagues [13] (*n *= 13,043) an etiology was confirmed in only 7% of patients. We acknowledge that the frequency of obtaining a microbiologic diagnosis is lower in our cohort than that in previous epidemiologic studies designed to assess the etiology of CAP [14]. The reasons for these differences are as follows: sputum studies were not obtained routinely in all patients and were collected in only one-third of our cohort; and serology studies for atypical infections and viruses were conducted in fewer than 5% of patients in our cohort. However, these practices are consistent with recommendations for diagnostic work up for CAP in the recent CAP guidelines [15].

The model developed here demonstrates that, for the purposes of simulating a cohort of individual patients with CAP over the course of their illness, a purely empiric strategy based entirely on the data available can reproduce the cohort-level characteristics of the data, yet provide direct and continuous information on the clinical condition of each individual progressing through the model. Such an approach can enhance the ability of investigators to develop clinically detailed and realistic simulation models for trial design, protocol development, and cost-effectiveness analysis. For example, one could use the model to predict the potential mortality and length of stay effect (and therefore effect size for a sample size calculation) of therapies designed to improve the function of various organs by simulating the effect of a discontinuous improvement in some component of the SOFA score. Simulation models have been commonly used in cost-effectiveness analysis, and the ability to recreate individual patient histories allows more detailed analysis of the source of costs in such models. Most of the examples of the use of individual simulation models are from other diseases [16,17], but we are building a platform to conduct such analyses in patients with sepsis. Furthermore, we and others have used these specific empiric techniques to address transplantation policy questions [18,19].

In future work we will extend the clinical description of patients to include more complex combinations of laboratory tests, physiologic state variables (blood pressure, pulse, among others), and genetic predispositions (presence or absence of certain polymorphisms) to represent more faithfully the clinical richness and complexity of patients with sepsis and organ failure.

## Conclusion

We used data from a large, multicenter study to develop a dynamic microsimulation model to predict disease progression in patients with pneumonia-related sepsis. The model is able to predict hospital discharges, in-hospital deaths, and serial SOFA scores of patients with sepsis, and it supports the assertion that the duration of disease is a critical factor in predicting the outcomes of sepsis.

## Key messages

• An empirically based simulation model can represent the clinical course and outcomes in sepsis, and reproduce severity of illness over time.

• The duration of illness is more important than the immediate acute change in illness in predicting future outcomes.

## Abbreviations

CAP = community-acquired pneumonia; ICU = intensive care unit; SOFA = Sepsis-related Organ Failure Assessment.

## Competing interests

The authors declare that they have no competing interests.

## Authors' contributions

GS conducted the majority of the programming and data analysis. JEK initiated the development of the simulation model and was responsible for the initial programming and development of the natural history component. AJS supervised GS and JEK and was responsible for ensuring the analytic accuracy of the model. CHC conducted the statistical significance tests in which the model's predictions were compared with the actual results. MSR provided intellectual oversight and development of the modeling components of this analysis, and completed much of the writing and editing. DCA provided critical care clinical oversight, access to the GenIMS database, and overall intellectual leadership of the clinical components of this work. All authors read and approved the final manuscript.

## Supplementary Material

Additional File 1 A Word document listing individuals and institutions participating in the GenIMS study, and adding further acknowledgements. Click here for file
