# Supplementary material for: Use of dynamic microsimulation to predict disease progression in patients with pneumonia-related sepsis
Source: Crit Care. 2007 Jun 14;11(3):R65. doi: 10.1186/cc5942 (PMC2206430; doi:10.1186/cc5942)
Supplement: Additional File 1 — A Word document listing individuals and institutions participating in the GenIMS study, and adding further acknowledgements. [file cc5942-S1.doc]

**Additional file 1**

The following individuals and institutions participated in the Genetic and Inflammatory Markers of Sepsis (GenIMS) Study. We are indebted to the nurses, respiratory ther­apists, phlebotomists, physicians, and other health care professionals, as well as the patients and their families, who supported this trial.

**Principal Investigator:** Derek C. Angus, MD, MPH

**Co-Principal Investigator:** John A. Kellum, MD

**Co-Investigators**

*University of Pittsburgh*—Vincent Arena, PhD; Gregory F. Cooper, MD, PhD; Russell L. Delude, PhD; Eleanor Feingold, PhD; Robert Ferrell, PhD; Michael J. Fine, MD, MSc; David Finegold, MD; Mitchell P. Fink, MD; Lan Kong, PhD; Alexander Krichevsky, DVM, PhD; Michael Martino, MD; Michael R. Pinsky, MD; Malik Rahim, MD; Mark S. Roberts, MD, MPP; Andrew Schaefer, PhD; Lisa A. Weissfeld, PhD; Kelly A. Wood, MD, MHS; Donald M. Yealy, MD. *Northwestern University*—Richard G. Wunderink, MD. *Norwalk Hospital*—Jonathan M. Fine, MD. *North Shore University Hospital*—Kevin J. Tracey, MD; Haichao Wang, PhD; LiHong Yang, PhD. *University of Pennsylvania*—Jason Christie, MD, PhD. *Yale University*—Richard Bucala, MD, PhD.

**Enrolling Study Sites**

Connecticut: Bridgeport Hospital—Michael Werdmann, MD; Kathy Nunnink, RN, BSN; Larry H. Bernstein, MD; Herbert Scherzer, MD; Raymond Haddad, MD. Hartford Hospital—Robert Grant, MD; Roseanne Papa, RN; Caryn St. Clair, RN; Beverly Reynholds, RN. Milford Hospital—Jay Walshon, MD; Tina Null, RN; Lloyd Friedman, MD; Suri Pappu, MD. New Britain General Hospital—Louis M. Graff, MD; Nancy Bennett, RN; David Buono, MD; Barry Jacobs, MD; Michael McNamme, MD; Stephen Wolf, MD. Norwalk Hospital—Michael Carius, MD; Christine Belden, RN; Leonard Scinto; Saraswathi Nair, MD. St. Mary’s Hospital—Steve Holland, MD; Eleanor Flynn, RN; Rose Riordan. Yale University Hospital—Linda C. Degutis, DrPH; Nancy Olson, RN, MS; Peter Jatlow, MD; Mark Siegel, MD.

Michigan: Detroit Receiving and Sinai Grace Hospitals—Robert Welch, MD; Denise Waselewsky, RN; Robert Dunne, MD; James Kruse, MD; Kristen Bilicki. Henry Ford Health System—Emanuel Rivers, MD; Kant Shah, MD.

Pennsylvania: Jefferson Regional Medical Center/South Hills Health System—Christopher Dooley, MD; Jacqueline Anderson, RN; David Laman, MD; Mahapareh Mostoufi, MD. Lee Regional Health System—Sandy Ergas, MD; Karen Betcher, RN; Antoinette Furman, RN; Ed Rocker. Mercy Hospital—Bruce MacLeod, MD; Susan Rolniak, RN, CRNP; Dennis Borochovitz, MD; Ken Greer, MD; Pat Riley, MD. Sewickley Valley Hospital—Frank Gaudio, MD; Greg Maggi, RN; Mindy Hufnagel. St. Clair Hospital—Christopher DeLuca, MD; Arlene Grogan, RN; Stephen Basheda, DO; Martha Clark, MD. The Western Pennsylvania Hospital—Thomas P. Campbell, MD; Diana Morrow, RN; Paul C. Fiehler, MD; Stanley Geyer, MD. University of Pittsburgh Medical Center (UPMC) Braddock—Richard Heath, MD; Sandra Casey, RN; Emily Yee, MD; Charles Krifcher, MD. UPMC Horizon Health System (Greenville and Shenango Hospitals)—Jeffrey Moldovan, DO; Janet Moldovan, RN, MSN; Amy Pagano, RN, MSN; Ed Castor. UPMC McKeesport—Rani K. Kumar, MD; Linda Hewitt, RN; Linda Knestaut, RN; Rahut Chaudhry, MD; Ray Probst. UPMC Passavant—William Kristan, MD; Linda Campbell, RN; Carol Hewlett, RN; Mark Provenzano, MD; Joe Kuzma. UPMC Presbyterian—Ted Delbridge, MD; Mary Ann Murcek, RN; Alan Wells, MD. UPMC Shadyside—Jerold Solot, DO; Linda Waddell, RN; Michael Becich, MD; Joel Weinberg, MD. UPMC South Side—Joel Rosenbloom, DO; Carol Joyce, RN; Sukamal Khasnabis, MD; C. Vaughn Strimlan, MD. UPMC St. Margaret—James Nicholas, MD; Ann Morris, RN; Nancy Gorsha, RN; Mitchell Patti, MD; Jagjit Singh, MD; Meredith Naples.

Tennessee: Methodist Healthcare (Methodist Le Bonheur Germantown, Methodist North, Methodist University Hospital)—Richard Wunderink, MD; Carol Jones, RN, BSN; Lori Kessler, PharmD.

**Clinical Coordinating Center Staff, CRISMA Laboratory, University of Pittsburgh**

Tammy L. Young; Margaret V. Bowman, RN, BSN; Melinda Carter; Jodi Gigler; Thomas E. Auble, PhD; Angela Darnley; Karen Greenwald, RN, BSN; Lynda J. Guzik; Susan Hebda, RRT; Tracy Hoteck, RN; Marcia McCaw, RN; Lee Anne Mandich, RN, BSN; Tricia A. Powell, RN, BSN; Angel Shaufl; Heather Sterling, RN; Heather Woods, RN, BSN.

**Data Coordinating Center Staff**

Javier Martinez, MBA/MoIS, PhD; Xia Tang, MSIS; Dara Green, MS; Shui He, MS; Jennifer Kreke, MS; Gorkem Saka, MS; Shyam Visweswaran, PhD; Xing Yuan.
